# Structural, elastic, mechanical and thermodynamic properties of HfB_4_ under high pressure

**DOI:** 10.1098/rsos.180701

**Published:** 2018-07-25

**Authors:** Jing Chang, Xiaolin Zhou, Ke Liu, Nina Ge

**Affiliations:** 1 College of Physics and Electronic Engineering, Sichuan Normal University, Chengdu 610068, People's Republic of China; 2 School of Materials Science and Engineering, Southwest Jiaotong University, Chengdu 610031, People's Republic of China; 3 State Key Laboratory Cultivation Base for Nonmetal Composites and Functional Materials, Southwest University of Science and Technology, Mianyang 621999, People's Republic of China

**Keywords:** first-principles, structural properties, mechanical properties, thermodynamic properties, HFB_4_

## Abstract

The present work aims to study the structural, elastic, mechanical and thermodynamic properties of the newly discovered orthorhombic *Cmcm* structure HfB_4_ (denoted as *Cmcm*-HfB_4_ hereafter) under pressure by the first-principles calculations. The obtained equilibrium structure parameters and ground-state mechanical properties were in excellent agreement with the other theoretical results. The calculated elastic constants and phonon dispersion spectra show that *Cmcm*-HfB_4_ is mechanically and dynamically stable up to 100 GPa and no phase transition was observed. An analysis of the elastic modulus indicates that *Cmcm*-HfB_4_ possesses a large bulk modulus, shear modulus and Young's modulus. The superior mechanical properties identify this compound as a possible candidate for a superhard material. Further hardness calculation confirmed that this compound is a superhard material with high hardness (45.5 GPa for GGA); and the relatively strong B–B covalent bonds’ interaction and the planar six-membered ring boron network in *Cmcm*-HfB_4_ are crucial for the high hardness. Additionally, the pressure-induced elastic anisotropy behaviour has been analysed by several different anisotropic indexes. By calculating the *B*/*G* and Poisson's ratio, it is predicted that *Cmcm*-HfB_4_ possesses brittle behaviour in the range of pressure from 0 to 100 GPa, and higher pressures can reduce its brittleness. Finally, the thermodynamic properties, including enthalpy (Δ*H*), free energy (Δ*G*), entropy (Δ*S*), heat capacity (*C_V_*) and Debye temperature (*Θ_D_*) are obtained under pressure and temperature, and the results are also interpreted.

## 1. Introduction

Ultra-incompressible and hard materials have attracted a great deal of attention owing to their outstanding physical and chemical properties in fundamental science and industry applications [1–4]. Previously, it was commonly accepted that superhard materials are those strong three-dimensional covalent compounds formed by light elements (B, C, N and O), such as diamond [5], c-BN [6], BC_2_N [7], B_6_O [8] and BC_5_ [9] etc. However, these superhard materials are infrequent and expensive because they can be synthesized only under extreme high-temperature and high-pressure conditions. To overcome the shortcoming, many research activities attempt to synthesize new intrinsically superhard materials by combining small strongly covalent bonding light elements and large electron-rich heavy transition metals (TMs). The compounds formed by TMs and light atoms usually possess high valence electron density and directional covalent bonds, which contribute to improve their mechanical properties and hardness. Based on this design principle, a series of ultra-incompressible and hard materials were successfully synthesized [6,10–16]. Recently, considerable attention has been paid to transition metal borides (TMBs), such as Re-B [3], Os-B [17], Ru-B [18] and W-B [19,20]. Especially the newly boron-rich TM compounds, such as WB_4_ [21,22], CrB_4_ [23,24], MnB_4_ [25–27], FeB_4_ [28,29], YB_4_ [30] and VB_4_ [31], have been extensively investigated. This may be due to boron having a strong ability to form covalent bonds with TMs. A lot of calculated results also show that all these materials exhibit excellent mechanical properties and high hardness. In addition, TMBs can be synthesized at ambient condition, which reduces the cost of synthesis and is beneficial for practical application. Therefore, in order to find new superhard materials, further studies are needed for the TMBs, especially the boron-rich TM compounds that have been proposed recently. The study will open up a new way for seeking new ultra-incompressible and hard materials.

In the boron-rich TM compounds, the studies of Hf-B compounds are surprisingly scarce, compared with the extensive research works on TMB_4_ (TM = W, Cr, Mn and Fe) [21–29]. For the Hf-B system, three known binary phases have been synthesized experimentally [32], namely HfB, HfB_2_ and HfB_12_. For HfB, earlier studies [33,34] suggested that it has an orthorhombic FeB structure and its thermal stabilization is in the range from 1200°C to 2000°C. A later experiment reported that HfB also has a NaCl-type phase [32]. With regard to HfB_2_, it possesses a hexagonal AlB_2_-type structure [32], exhibits many unique properties, such as high chemical and thermodynamic stability, high hardness and low electrical resistivity; these characteristics make HfB_2_ become a promising candidate for various high-temperature structure materials [35]. For the HfB_12_, its cubic structure was experimentally synthesized at 6.5 GPa and 1600–2100°C [36]. Recently, an experimental study showed that the cubic structure of HfB_12_ can be successfully stabilized in the Y_1−x_Hf_x_B_12_ system under ambient pressure [37]. Most recently, a novel promising phase, orthorhombic *Cmcm* structure HfB_4_, has been proposed by Zhang *et al*. [38] with the particle swarm optimization (PSO) algorithm. The calculated results show that the *Cmcm*-HfB_4_ may be a potential promising superhard material and energetically much more stable than the previously proposed YB_4_-, ReP_4_-, FeB_4_-, CrB_4_- and MnB_4_-type structures at zero pressure. However, up to now, there are few works in the literature reporting on the physical properties of *Cmcm*-HfB_4_ under pressure (to our knowledge). Moreover, many fundamental aspects are still not well understood because of its complex structural and chemical behaviour. Therefore, a detailed study of the physical properties of *Cmcm*-HfB_4_ is undoubtedly necessary and may bring new insight for further understanding its unique mechanical properties.

In this study, we studied the structural, elastic and mechanical properties of the novel *Cmcm*-HfB_4_ under ambient and high pressures by the first-principles approach based on density functional theory (DFT). The effects of pressure and temperature on the thermodynamic properties of HfB_4_ are systematically obtained. This paper is organized as follows. In §2, the methods used are described. In §3, the main results of our calculations of structural and mechanical properties under pressure are given and compared with other TMBs. The computed thermodynamic properties are also reported in this section. Finally, in §4, the conclusions of this work are given.

## 2. Computational methods and details

In the current work, all theoretical calculations were carried out by the first-principles plane-wave pseudo-potential method based on DFT in Cambridge Serial Total Energy Package (CASTEP) code [39]. The contribution of core electrons is described using the Vanderbilt non-local ultrasoft pseudopotential [40]. Pseudo-atom calculations are performed for B: 2s^2^2p^1^ and Hf: 5p^6^5d^2^6s^2^. To compare the performance of different approximations of exchange–correlation interaction, here we used the generalized gradient approximation (GGA) with the Perdew–Burke–Ernzerhof (PBE) functional [41] and the modified PBE functional (PBESOL) [42], which was proved to provide good results for solids of high density [43], as well as the local density approximation (LDA) with the form of Ceperley–Adler parametrized by Perdew & Zunger (CAPZ) [44], used to describe the exchange–correlation potentials. The structures were relaxed using the Broyden, Fletcher, Goldfarb and Shannon (BFGS) minimization method algorithm [45]. The lattice constants and atom coordinates were optimized by minimizing the total energy. Through a series of convergence studies with respect to cut-off energies and *k*-points, the cut-off energies were set at 500 eV, and the K-space integration over the Brillouin zone was carried out using a 5 × 8 × 2 *k*-point Monkhorst-Pack mesh for the compounds. The values of the kinetic energy cut-off and the *k*-point density were determined to ensure the convergence of total energies to within 0.01%. In geometrical relaxations, the residual force is less than 0.001 eV Å^−1^, the maximum displacement of atoms to less than 5.0 × 10^−4^ Å and the convergence threshold for the maximum stress to 0.02 GPa. All these parameters were tested to ensure that the self-consistent total energies converged to within 5.0 × 10^−7^ eV atom^−1^.

The elastic constants *C_ij_*, needed for the calculation of mechanical properties and to study the mechanical stability of *Cmcm*-HfB_4_, were calculated through *C_ij_* = *σ_i_*/*ε_j_*, where *σ* and *ε* are the elastic stress and strain, respectively. The subscripts *i* and *j* denote the Cartesian coordinates of the considered structures [46]. For the optimization of the internal atomic freedoms, the criteria for convergence were set as follows: the difference in total energy was within 1.0 × 10^−6^ eV atom^−1^, maximum force was 0.002 eV Å^−1^, maximum displacement was 1.0 × 10^−4^ Å and maximum stress within 100 GPa. Based on these elastic constants and Voigt–Reuss–Hill (VRH) approximations, the bulk modulus (*B*), shear modulus (*G*), Young's modulus (*E*) and Poisson's ratio (*ν*) were derived by the formulae: *E *= 9*BG*/(3*B *+ *G*) and *ν *= (3*B *− 2*G*)/[2(3*B *+ *G*)]. The effective bulk and shear moduli are also introduced by *B *=* *(*B_V_ *+* B_R_*)/2 and *G *=* *(*G_V_ *+* G_R_*)/2, where the subscripts *V* and *R* denote the Voigt and Reuss approximation, respectively.

The phonon dispersion curves and phonon density of states (DOS) were determined by using the linear response density functional perturbation theory [47,48]. The phonon frequencies in the Brillouin zone (BZ) centre are computed as second-order derivatives of the total energy with respect to atomic displacements. In addition, some phonon-related thermodynamic properties such as the enthalpy (Δ*H*), entropy (Δ*S*), free energy (Δ*G*), lattice heat capacity (*C_v_*) and Debye temperature (*Θ_D_*) are evaluated in a quasi-harmonic approximation.

## 3. Results and discussion

### 3.1. Structural properties under pressure

**Figure 1.**
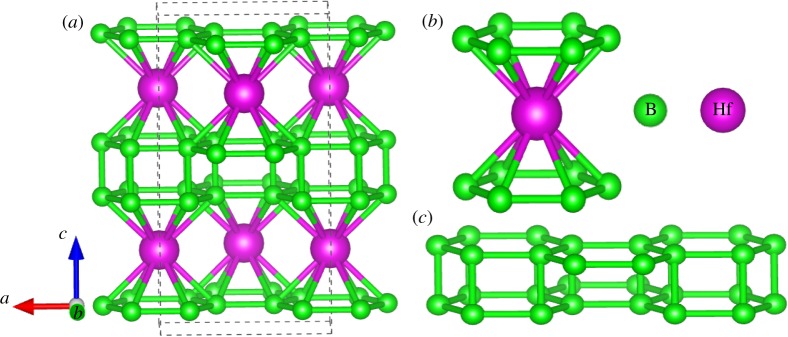
(*a*) Optimized energetically most stable crystal structures of *Cmcm*-HfB_4_ at zero pressure; (*b*) HfB_12_ polyhedron and (*c*) B_4_ unit chain in the structure.

The crystal structure of HfB_4_ is orthorhombic with the symmetry of the space group *Cmcm* (no. 63, figure 1*a*–*c*). The *Cmcm*-HfB_4_ contains four HfB_4_ formula units (f.u.) in its unit cell, in which two inequivalent Hf and B atoms occupy the Wyckoff 4c (0, 0.4190, 0.75) and 16h (0.8312, 0.8767, 0.5795) position, respectively. Surprisingly, in *Cmcm* phase, each metal Hf atom is surrounded by 12 neighbouring B atoms, and B atoms form parallel hexagonal planes, which results in the formation of HfB_12_ hexagonal columns (as shown in figure 1*b*). These structural units form intriguing B_6_–Hf–B_6_ sandwiches stacking order along the crystallographic *c*-axis, which exhibit ultra-incompressible characterization. In the HfB_12_ polyhedron, the three types of Nb-B bond distances are calculated to be 2.448 (× 4), 2.505 (× 4) and 2.612 (× 4) Å. In the quadrangle B ring, the shortest B–B bond distance is 1.7791 Å, which is smaller than that of the known hard materials ReB_2_ (1.81 Å), OsB_2_ (1.85 Å) and Os_2_B_3_ (1.876 Å), indicating that the *Cmcm*-HfB_4_ structure may have strong stability and good mechanical properties.

To obtain a stable geometry structure, we first make the structural relaxation and optimization with the two different exchange–correlation approximations (GGA and LDA), to determine the internal atomic coordinates and structure parameters. The obtained lattice parameters, density, unit cell volume and atomic positions within the GGA and LDA are listed in table 1 together with the available results and similar TMBs for comparison. From table 1, it can be seen that the obtained equilibrium lattice constants, density, cell volumes and atomic positions at the GGA/LDA level are also in excellent agreement with the theoretical values [31,49]. This good agreement shows the accuracy of our calculations. In addition, the results from GGA (PBE and PBESOL) have a better agreement with the previous data compared to the LDA (CAPZ), owing to the obtained lattice parameters by the two exchange correlation functions (PBE and PBESOL) being almost same for the *Cmcm*-HfB_4_ compound. Therefore, in this work, we only choose the GGA-PBE method in the following calculations.

**Table 1.** Optimized equilibrium lattice parameters *a* (Å), *b* (Å) and *c* (Å), density *ρ* (g cm^−3^), cell volume *V* (in Å^3^) and atomic position of *Cmcm*-HfB_4_ at zero pressure, together with other TMBs.

structure	this study	*a*	*b*	*c*	*ρ*	*V*	atom position
*Cmcm*-HfB_4_	GGA-PBE	5.359	3.136	10.361	8.458	174.132	Hf(4c) 0, 0.4190, 0.75
							B(16h) 0.8312, 0.8767, 0.5795
	GGA-PBESOL	5.359	3.139	10.359	8.452	174.242	Hf(4c) 0, 0.4184, 0.75
							B(16h) 0.8309, 0.8758, 0.5786
	LDA-CAPZ	5.293	3.098	10.20	8.803	167.301	Hf(4c) 0, 0.4175, 0.75
							B(16h) 0.8311, 0.8763, 0.5799
	[38]	5.360	3.134	10.356	8.466	173.963	Hf(4c) 0, 0.4189, 0.75
							B(16h) 0.8309, 0.8766, 0.5796
*Cmcm*-ZrB_4_	GGA-PBE	5.376	3.146	10.461	5.048	176.933	Zr(16f) 0, 0.9191, 0.75
							B(4c) 0.1690, 0.3750, 0.5779
	GGA-PBESOL	5.363	3.138	10.406	5.100	175.125	Zr(16f) 0, 0.9159, 0.75
							B(4c) 0.1690, 0.3742, 0.5783
	LDA-CAPZ	5.306	3.105	10.316	5.254	169.970	Zr(16f) 0, 0.9165, 0.75
							B(4c) 0.1691, 0.3739, 0.5779
	[49]	5.376	3.145	10.466	5.047	176.954	Zr(16f) 0, 0.9194, 0.75
							B(4c) 0.1695, 0.3749, 0.5776
*Cmcm*-VB_4_	GGA-PBE	4.885	10.630	2.951	4.082	153.237	B1(8g) 0.3141, 0.0436, 0.75
							B2(8g) 0.2997, 0.2074, 0.75
							V(4c) 0, 0.1130, 0.25
	GGA-PBESOL	4.854	10.614	2.941	4.128	151.530	B1(8g) 0.3132, 0.0437, 0.75
							B2(8g) 0.2996, 0.2073, 0.75
							V(4c) 0, 0.11286, 0.25
	LDA-CAPZ	4.808	10.501	2.908	4.260	146.837	B1(8g) 0.3134, 0.0436, 0.75
							B2(8g) 0.2996, 0.2073, 0.75
							V(4c) 0, 0.11283, 0.25
	[31]	4.889	10.616	2.964	4.066	153.836	B1(8g) 0.3134, 0.0435, 0.75
							B2(8g) 0.3, 0.2076, 0.75
							V(4c) 0, 0.11303, 0.25

**Figure 2.**
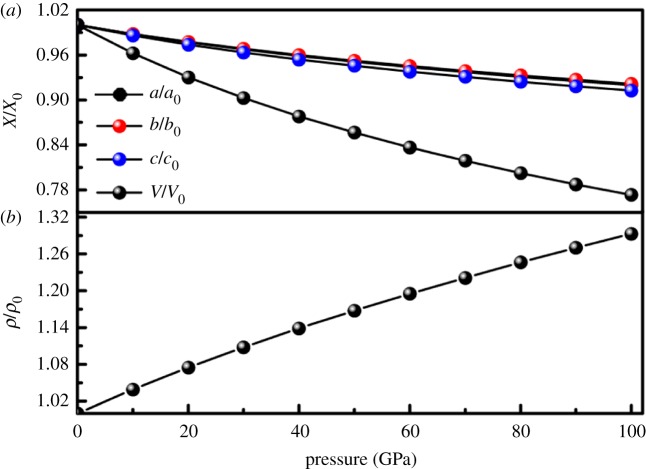
The normalized parameters *X/X*_0_(*X* = *a*, *b*, *c* and *V*), *ρ/ρ*_0_ of *Cmcm*-HfB_4_ as a function of pressure.

As is well known, pressure can change the structural stability and mechanical behaviour of a compound during its synthesis process. To provide some insight into the pressure behaviour of HfB_4_, the normalized parameters *a/a*_0_, *b/b*_0_, *c/c*_0_ and volume *V/V*_0_ as a function of pressure are shown in figure 2, where *a*_0_, *b*_0_, *c*_0_ and *V*_0_ are the equilibrium structural parameters at zero pressure, respectively. From figure 1*a*, it is obvious that the values of the structural parameters decrease with increasing pressure, which means that the bond length of *Cmcm*-HfB_4_ becomes shorter as the pressure increases. Meanwhile, the curves also present that the crystal cell of *Cmcm*-HfB_4_ along the crystallographic *b*-axis is much more difficult to compress than in the other directions in the whole range of pressure. This may be due to the fact that there is very strong local covalent bonding between Hf atoms and B atoms. In addition, figure 2 shows that pressure effects will generally reduce the volume and increase the density of *Cmcm*-HfB_4_. Moreover, in the pressure range studied here, no transition between the different phases occurs.

### 3.2. Elastic and mechanical properties under pressure

The elastic properties (e.g. elastic constants, elastic moduli and elastic anisotropy etc.) under pressure are of key importance for us to understand the deformation behaviour of a solid in response to external forces, and can provide a deeper insight into macroscopic mechanical behaviour and help estimate the hardness of materials. Therefore, an explicit determination of the elastic properties of *Cmcm*-HfB_4_ as a function of pressure is essential, and will have an important guidable significance to accelerate the synthesis of *Cmcm*-HfB_4_. The predicted zero pressure elastic constants *C_ij_*, nine for the orthorhombic phase (*C*_11_, *C*_22_, *C*_33_, *C*_44_, *C*_55_, *C*_66_, *C*_12_, *C*_13_ and *C*_23_) of *Cmcm*-HfB_4_ are determined by the strain–stress method. These results are listed in table 2 along with other typical TMBs (TMB_4_, TM = Y, Fe, Cr, Mn, Ta, Zr, Mo, Re, V and Os) for comparison. From table 2, our results are in reasonable agreement with those obtained by Zhang *et al*. [38]. The good agreement between the current and previous results supports the accuracy and reliability of our elastic calculations.

**Table 2.** Calculated elastic constants *C*_ij_ (GPa) for *Cmcm*-HfB_4_ at zero pressure, together with other TMBs.

structure	this study	*C*_11_	*C*_22_	*C*_33_	*C*_44_	*C*_55_	*C*_66_	*C*_12_	*C*_13_	*C*_23_
*Cmcm*-HfB_4_	GGA-PBE	606	604	501	247	194	262	53	77	97
	LDA-CAPZ	650	648	544	270	213	278	59	89	109
	[38]	606	611	505	251	197	264	53	83	99
*Cmcm*-ZrB_4_	GGA-PBE	579	582	487	238	193	251	41	87	101
	[49]	555	560	493	223	199	261	65	90	96
*Cmcm*-VB_4_	GGA-PBE	568	742	587	268	49	160	85	83	93
	[31]	547	736	567	266	90	163	83	62	89
*Pmmn*-OsB_4_	[50]	612	516	630	152	349	178	128	245	
*P4/mbm*-YB_4_	[51]	454		442	108		132	80	33	
*P63/mmc*-MoB_4_	[52]	567		936	189			141	129	
*Immm*-CrB_4_	[23]	591	931	467	252	280	225	64	115	97
*C2/m*-TaB_4_	[53]	497	571	530	242	238	210	139	130	97
*P63/mmc*-ReB_4_	[54]	567		987	187			111	129	
	[23]	381	710	435	218	114	227	137	143	128

Next, we consider the mechanical stability, which is a necessary condition for the stability of crystal. For an orthorhombic crystal, the requirement for mechanical stability obeys the following restrictions on the elastic constants: [55] *C_ij_* > 0 (*i* = 1–6), (*C*_11_ + *C*_22_ − 2*C*_12_) > 0, (*C*_11_ + *C*_33_ − 2*C*_13_) > 0, (*C*_22_ + *C*_33_ − 2*C*_23_) > 0 and [*C*_11_ + *C*_22_ + *C*_33_ + 2(*C*_12_ + *C*_13_ + *C*_23_)] > 0. Our calculated results show that the obtained elastic constants of *Cmcm*-HfB_4_ under pressure satisfy the mechanical stability criteria, implying that the *Cmcm*-HfB_4_ is mechanically stable up to 100 GPa.

The polycrystalline bulk modulus (*B*) and shear modulus (*G*) of *Cmcm*-HfB_4_ are thus determined by the VRH approximation. The calculated bulk modulus, shear modulus, Young's modulus and Poisson's ratio of the *Cmcm*-HfB_4_ together with the reference materials mentioned above are also listed in table 3. From table 3, one can find that the presented elastic moduli are consistent with that of [38] and the LDA results are larger than the GGA ones. Generally, a large elastic modulus means high hardness of materials. In this work, the obtained elastic moduli are comparable to those of known hard materials, indicating that this structure possesses strong ability to resist volume deformation and is difficult to compress. However, only high bulk modulus is not enough to ensure high hardness; the shear modulus of materials, which is a measure of the ability to resist shape change at a constant volume, plays a more important role in hardness than the bulk modulus of materials. From table 3, it is found that *Cmcm*-HfB_4_ has a much larger shear modulus (237 and 255 GPa for GGA and LDA, respectively) than TMB_4_ (TM = Y, Fe, Mn, Ta, Zr, Mo, Re, Os, and V) [31,49–54].

**Table 3.** Calculated bulk modulus *B* (GPa), shear modulus *G* (GPa), Young's modulus *E* (GPa), Poisson's ratio *ν*, compressibility coefficient *β*, *B*/*G* ratio and hardness *H_v_* of *Cmcm*-HfB_4_ at zero pressure together with other TMBs.

structure	this study	*B*	*G*	*E*	*ν*	*β*	*G*/*B*	*H_v_*
*Cmcm*-HfB_4_	GGA-PBE	239	237	535	0.1276	0.0042	0.992	45.5
	LDA-CAPZ	261	255	577	0.1315	0.0038	0.977	46.7
	[38]	243	240	542	0.128		0.987	45.7
*Cmcm*-ZrB_4_	GGA-PBE	234	229	518	0.131	0.0042	0.98	43.8
	[49]	236	226	515	0.139		0.96	42.8
*Cmcm*-VB_4_	GGA-PBE	268	168	416	0.240	0.00376	0.63	
	[31]	255	193	462	0.20			
*Pmmn*-OsB_4_	[50]	294	218	514	0.294			
*P4/mbm*-YB_4_	[51]	182	144	342	0.18		0.791	17.04
*P63/mmc*-MoB_4_	[52]	285	210	506	0.204			
*Immm*-CrB_4_	[23]	275			0.142		0.942	45.1
*C2/m*-TaB_4_	[53]	259	220	515	0.17		0.855	29
*P63/mmc*-ReB_4_	[54]	317	223	560	0.206			

This suggests that *Cmcm*-HfB_4_ may be a potential superhard material. In addition, the Young's modulus is also an important parameter for measuring the stiffness of a material in addition to the bulk modulus and shear modulus. The larger the Young's modulus a material has, the harder it is to deform. From table 3, the Young's modulus (535 GPa for GGA and 577 GPa for LDA) of HfB_4_ is larger than the other compounds [31,49–52], indicating that HfB_4_ has a higher hardness than the other TM compounds. All of these excellent mechanical properties strongly suggest that m-HfB_4_ is a potential candidate for a superhard material.

Owing to the large elastic constants, and high bulk and shear moduli for *Cmcm*-HfB_4_, its hardness calculation is of great importance. It denotes the resistance to elastic and plastic deformations when a force is loaded. Here, we predicted the hardness of *Cmcm*-HfB_4_, which can be correlated with the product of the squared Pugh's modulus ratio and the shear modulus. The Vickers hardness is estimated by [56]

3.1 $$H_{v}=2{\left(K^{2}G\right)}^{0.585}-3,$$

where *K* represents the *G*/*B* ratio and *G* is the shear modulus. Using the model, the estimated Vickers hardness *H*_v_ of *Cmcm*-HfB_4_ is in good agreement with a previous study [38]. The high hardness of *Cmcm*-HfB_4_ probably stems from the relatively strong B–B covalent bonding and the presence of a planar boron six-membered ring unit in *Cmcm*-HfB_4_ structure.

**Figure 3.**
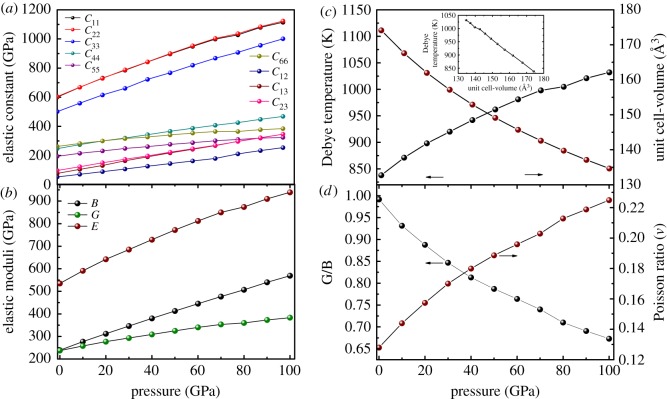
(*a*) Elastic constants, (*b*) elastic moduli, (*c*) Debye temperature *Θ_D_*—unit cell volume and (*d*) variation of the G/B—Poisson ratio (*ν*) of the *Cmcm*-HfB_4_ compounds under pressure. The inset in (*c*) shows the Debye temperature as a function of the unit cell.

In addition, one should be aware that the values presented in tables 2 and 3 were obtained at *P *= 0 GPa, and that pressure effects will generally increase the elastic constants and mechanical modulus. To further explore the influence of pressure on the elastic and mechanical properties of *Cmcm*-HfB_4_, one can use the relationship between the elastic constants and pressure shown in figure 3. Unfortunately, there are no literature data to compare with our predicted results for the pressure derivative of elastic properties of *Cmcm*-HfB_4_. Therefore, our results can serve as a prediction for future studies. From figure 3*a*,*b*, clearly, the elastic constants and the elastic modulus monotonically increase under pressure. The elastic constants are positive and satisfy the well-known Born criteria [57], indicating that *Cmcm*-HfB_4_ is a mechanically stable phase up to *P*= 100 GPa. From figure 3*a*, it is clear that the calculated *C*_22_ value is higher than those of *C*_11_ and *C*_33_, implying that the resistance to deformation along the *b*-axis is stronger than that along the *a*- and *c*-axis. This just corresponds with the above-determined fact that it is much more difficult to be compressed along the *b-axis* than along the *a-* and *c*-*axis* under pressure. In addition, *C*_44_ is an important indicator for the hardness of a material. The large *C*_44_ value (247 GPa for GGA and 270 for LDA) indicates its relatively strong strength against shear deformation. From figure 3*b*, it can be seen that the elastic moduli monotonically increase under pressure, suggesting that *Cmcm*-HfB_4_ is more difficult to compress with increase in pressure.

To further probe into the physical properties of *Cmcm*-HfB_4_, we analysed its ductility and brittle nature under pressure according to the Pugh criterion [58]. A material with a high *G*/*B* ratio value (greater than 0.57) is associated with brittle nature; otherwise, materials should have ductility. Our results show that the *G*/*B* ratio changes from 0.99 (*P *= 0 GPa) to 0.67 (*P *= 100 GPa). These values indicate that *Cmcm*-HfB_4_ shows behaviour as a brittle material in the whole pressure range from 0 to 100 GPa (figure 3*d*). The relative directionality of the bonding in the material also has an important effect on its hardness and can be determined from the *G*/*B* ratio. HfB_4_ at 0 GPa has the largest *G*/*B* ratio, which suggests the strongest directional bonding between the Hf and B atoms, and this bonding decreases as the pressure increases.

The calculated Poisson ratio (*ν*) is an additional argument used to describe the ductility and brittle nature of materials, with 0.33 as the critical reference point. For brittle metallic materials, these values are small, whereas for ductile metallic materials, the value is bigger than 0.33. In this study, *ν* changes from 0.13 (*P *= 0 GPa) to 0.22 (*P *= 100 GPa); therefore, *Cmcm*-HfB_4_ shows behaviour as a brittle material and then it tends to become ductile (figure 3*d*). In addition, the *ν* value is far below 0.33, suggesting a high degree of covalent bonding, which contributes to the material's hardness. The Poisson's ratio of *Cmcm*-HfB_4_ (0.128 for GGA) is smaller than that of the previously reported hard material ReB_2_ (0.171), indicating that the degree of covalent bonding of *Cmcm*-HfB_4_ is very good because of the presence of a planar six-membered ring boron network. The Debye temperature *Θ_D_* as a function of pressure is shown in figure 3*c*. Moreover, the calculated *Θ_D_* value shows a linear increase with increase in pressure due to stiffening of the crystal structure.

**Figure 4.**
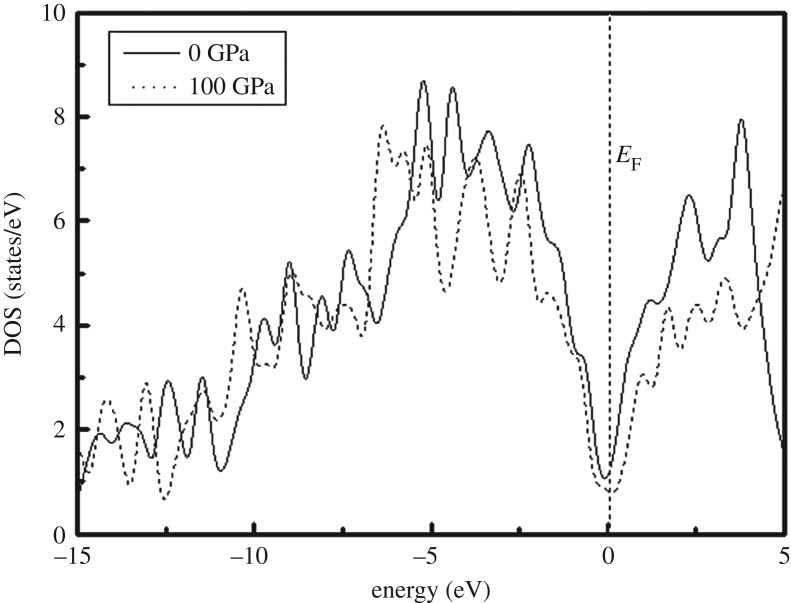
Calculated DOS of the *Cmcm*-HfB_4_ at 0 and 100 GPa. The vertical dashed line shows the Fermi level *E*_F_.

To further understand the mechanical properties of *Cmcm*-HfB_4_ under pressure, we calculated the DOS at 0 and 100 GPa, and the corresponding results are shown in figure 4. The Fermi energy level was taken as the origin of the energy. As is clearly shown in figure 4, *Cmcm*-HfB_4_ presents a metallic character. There is a deep valley and significant pseudo-gap at *E*_F_, which indicates the covalent interaction between Hf and B of *Cmcm*-HfB_4_. In addition, the pseudo-gap is widened with the increase in pressure, revealing that the bond length is reduced under pressure, while the covalent interaction between Hf and B is increased with the increase in pressure. Therefore, this is also the reason for the high elastic modulus and the high hardness of *Cmcm*-HfB_4_.

### 3.3. Elastic anisotropy under pressure

Considering that the microcracks are easily induced in materials owing to significant elastic anisotropy especially for hard materials, it is worthwhile to investigate the elastic anisotropy of materials. The shear anisotropic factors provide a measure of the degree of anisotropy in the bonding between atoms in different planes. The shear anisotropic factor for the {100} shear planes between the ⟨011⟩ and ⟨010⟩ directions is

3.2 $$A_{1}=\frac{4C_{44}}{C_{11}+C_{33}-2C_{13}}.$$

For the {010} shear planes between the ⟨101⟩ and ⟨001⟩ directions, it is

3.3 $$A_{2}=\frac{4C_{55}}{C_{22}+C_{33}-2C_{23}}.$$

And for the {001} shear planes between the ⟨110⟩ and ⟨010⟩ directions, it is

3.4 $$A_{3}=\frac{4C_{66}}{C_{11}+C_{22}-2C_{12}}.$$

For orthorhombic crystals, the elastic anisotropy arises from the anisotropy of the linear bulk modulus in addition to the shear anisotropy. The anisotropies of the bulk modulus along the *a*-axis and the *c*-axis with respect to the *b*-axis can be written as

3.5 $$A_{B_{a}}=\frac{B_{a}}{B_{b},A_{B_{c}}}=\frac{B_{c}}{B_{b}},$$

where *B_a_, B_b_* and *B_c_* are the bulk moduli along different crystal axes, defined as

3.6 $$B_{i}=i\frac{\text{d}P}{\text{d}i},i=a,b,c.$$

For an isotropic crystal, these factors must be 1.0. Any departure from 1.0 is a measure of the elastic anisotropy of the crystal. A more practical measure of elastic anisotropy is defined as follows:

3.7 $$A_{B}=\frac{B_{V}-B_{R}}{B_{V}+B_{R}},\;A_{G}=\frac{G_{V}-G_{R}}{G_{V}+G_{R}},$$

where *B* and *G* denote the bulk and the shear modulus, and the subscripts *V* and *R* represent the Voigt and Reuss approximations, respectively. A value of zero is associated with elastic isotropy, while a value of one indicates the largest possible elastic anisotropy. The calculated anisotropic factors under different pressure are collected in table 4. For *Cmcm*-HfB_4,_ *A_B_* = 0.18%, *A_G_* = 0.8% at 0 GPa, indicating that it is nearly isotropic. The percentage of bulk modulus anisotropy is smaller than the percentage of shear modulus anisotropy under pressure, suggesting that there is less anisotropic incompressibility than in shear. In addition, the compressibility anisotropy factor $A_{B_{b}}$ is nearly equal to 1.0, indicating that the compressibility is almost the same along the *a-* and *b*-directions, which is in accordance with the pressure dependence of the normalized parameters *a*/*a*_0_ and *b*/*b*_0_. This phenomenon can be understood by the knowledge of the bonding situations in *Cmcm*-HfB_4_, which are characterized as the strong chemical bonding between the Hf and the B atoms. When pressure increases, the atoms in the HfB_4_ compound become closer, and their interactions become stronger to play an important role to form a superhard material. These mechanical properties also imply the anisotropy of HfB_4_.

**Table 4.** The pressure (in GPa) dependence of the shear anisotropic factors *A_1_*, *A_2_*, *A_3_*, anisotropy of bulk modulus *A_B_* and *A_G_* (in %), and compressibility anisotropy factors *A_Ba_* and *A_Bc_* of *Cmcm*-HfB_4_.

*P*	*A_1_*	*A_2_*	*A_3_*	*A_B_*	*A_G_*	*A_Ba_*	*A_Bc_*
0	1.042	0.851	0.953	0.00183	0.00866	0.957	0.843
10	1.081	0.875	0.943	0.00140	0.00827	0.963	0.852
20	1.107	0.884	0.935	0.00103	0.00837	1.083	0.969
30	1.144	0.900	0.930	0.00085	0.00824	0.973	0.882
40	1.159	0.890	0.912	0.00052	0.00822	0.977	0.895
50	1.191	0.903	0.903	0.00043	0.00843	0.980	0.907
60	1.207	0.900	0.897	0.00035	0.00881	0.983	0.918
70	1.223	0.903	0.888	0.00024	0.00909	0.986	0.927
80	1.269	0.917	0.889	0.00018	0.00905	0.987	0.935
90	1.288	0.912	0.889	0.00014	0.00956	0.990	0.942
100	1.314	0.905	0.887	0.00008	0.01034	0.991	0.947

**Figure 5.**
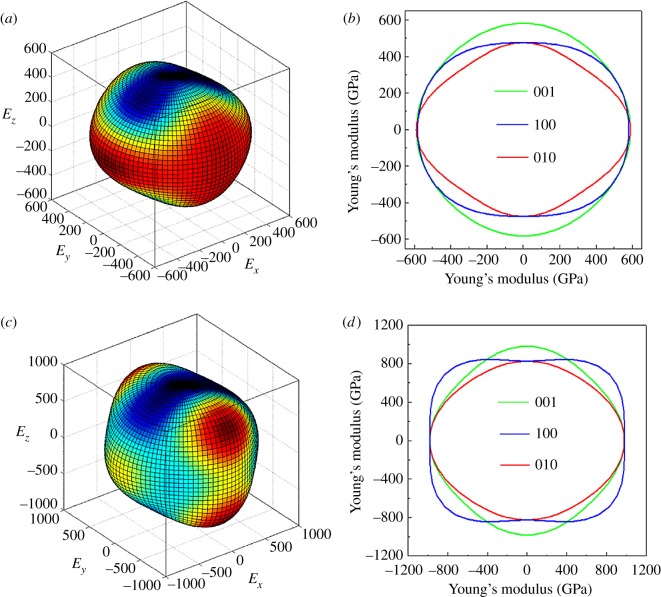
(*a*–*d*) Direction-dependent Young's modulus (GPa) and its plane projections for *Cmcm*-HfB_4_ at 0 (*a*,*b*) and 100 GPa (*c*,*d*).

To better understand the anisotropic behaviour, we obtained the 3D surface representations of the direction-dependent Young's modulus and its plane projections at 0 and 100 GPa, as shown in figure 5*a*–*d*). From figure 5, one can discover that the (001) plane exhibits isotropic property but the (100) and (010) planes exhibit anisotropic behaviour at 0 GPa; these results are consistent with those obtained by Zhang *et al*. [38] In addition, from figure 5*c*,*d*, it is not difficult to find that the anisotropy is significant with increase in pressure, which is in accordance with the above analysis from the anisotropy factor.

### 3.4. Thermodynamic properties under pressure

At present, most of the studies on the relative stability of polymorphs mainly consider the minimum lattice energies at 0 K and 0 bar condition. [59,60] However, considering the influence of the pressure and temperature on the structure, it is necessary to investigate the thermodynamic properties, such as the Gibbs free energy (Δ*G*), which can be used to determine the relative stability of polymorphs at desired thermodynamic conditions [43]. In addition, theoretical studies of the thermal properties are complementary to experiments and are useful for extending the regions of phase space which cannot be reached experimentally. Moreover, considering the pressure can change the mechanical stability and the reaction kinetics of a compound during its synthesis process, an explicit determination of its thermodynamic properties under pressure is essential.

Figure 7*a* shows the enthalpy, free energy and entropy under pressure. The Helmholtz free energy, *F* = *E* – T*S (where *F* is the free energy) is the relevant potential in an ensemble, where the volume and temperature are independent variables. As shown in figure 7*a*, below 100 K, the values of the enthalpy, free energy and entropy are almost zero. Above 100 K, the free energy at 0.0 GPa decreases faster with increasing temperature with respect to 100 GPa, and the TS at 0.0 GPa increases rapidly as the temperature increases with respect to 100 GPa, resulting in a linearly increasing relationship between the variations of enthalpy. Figure 7*b* shows the variations of the lattice heat capacity *C_V_* with temperature. We observe that the *C_V_* increases with the applied temperature but decreases under pressure. Below a temperature of approximately 900 K, *C_V_* increases very rapidly with temperature; above 900 K, *C_V_* increases slowly and gradually approaches the Dulong–Petit limit.

**Figure 6.**
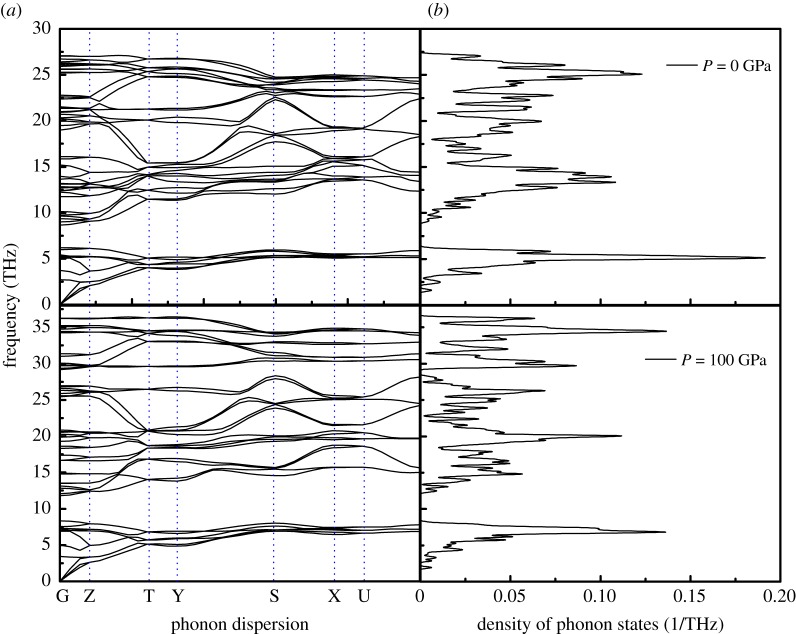
(*a*) Calculated phonon dispersion curves along the high-symmetry directions; and (*b*) density of phonon states for *Cmcm*-HfB_4_ at *P *= 0 and 100 GPa.

**Figure 7.**
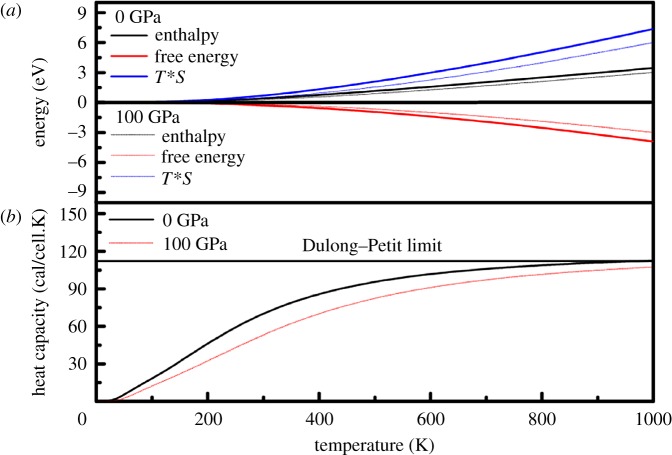
(*a*) Temperature dependence of enthalpy, free energy and T*S; and (*b*) heat capacity dependence of temperature for *Cmcm*-HfB_4_ at *P* = 0 and 100 GPa.

## 4. Conclusion

In summary, a systematic investigation of the structural, elastic, mechanical and thermodynamic properties of *Cmcm*-HfB_4_ under pressure has been performed by means of the *ab initio* plane wave pseudo-potential DFT method. The optimized lattice parameters are in excellent agreement with other theoretical results. The effect of pressure on the lattice normalized parameters shows that it is much more difficult to compress along the *b*-axis than in the other directions in the whole range of pressure. An analysis of the elastic calculated results show that the obtained elastic constants are strongly pressure-dependent, and the structure remains mechanically stable with brittle behaviour under pressure up to 100 GPa. In addition, an analysis of the elastic modulus shows that *Cmcm*-HfB_4_ possesses superior mechanical properties. The hardness calculation confirmed that *Cmcm*-HfB_4_ is a superhard material (*H_v_* > 40 GPa); and the presence of relatively strong B–B covalent bonds’ interaction and a planar six-membered ring boron network in *Cmcm*-HfB_4_ are crucial for its high hardness. Finally, its thermodynamic properties, including enthalpy, free energy, entropy, heat capacity and Debye temperature were obtained as a function of pressure and temperature. Based on them, we confirm that the reported *Cmcm* structure HfB_4_ is stable up to 100 GPa. We hope that high-pressure experiments will be done on synthesis of this superhard material for applications.

## Data accessibility

This article has no additional data.
